# Supplementary figures and images for: Automated image segmentation method to analyse skeletal muscle cross section in exercise-induced regenerating myofibers
Source: Sci Rep. 2021 Oct 29;11:21327. doi: 10.1038/s41598-021-00886-3 (PMC8556272; doi:10.1038/s41598-021-00886-3)

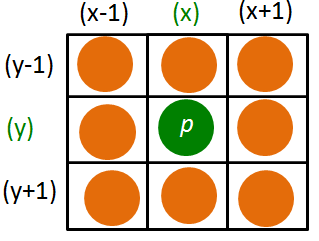


**Figure S1.** 8-neighbor used to find connected components.

Supplement: Supplementary file 1 — Supplementary Information. [file 41598_2021_886_MOESM1_ESM.docx]
